# Three-dimensional cardiovascular imaging-genetics: a mass univariate framework

**DOI:** 10.1093/bioinformatics/btx552

**Published:** 2017-09-04

**Authors:** Carlo Biffi, Antonio de Marvao, Mark I Attard, Timothy J W Dawes, Nicola Whiffin, Wenjia Bai, Wenzhe Shi, Catherine Francis, Hannah Meyer, Rachel Buchan, Stuart A Cook, Daniel Rueckert, Declan P O’Regan

**Affiliations:** 1 Department of Computing, Imperial College London, South Kensington Campus, London, UK; 2 Cardiovascular Magnetic Resonance Imaging and Genetics, MRC London Institute of Medical Sciences, Imperial College London, Hammersmith Hospital Campus, London, UK; 3 Quantitative Physiology and Genetics, National Heart and Lung Institute, Imperial College London, London, UK; 4 NIHR Cardiovascular Biomedical Research Unit, Royal Brompton and Harefield NHS Trust, London, UK; 5 European Molecular Biology Laboratory (EMBL), European Bioinformatics Institute, Wellcome Trust Genome Campus, Hinxton, UK; 6 Department of Cardiology, National Heart Centre Singapore, Singapore; 7 Programme in Cardiovascular and Metabolic Disorders, Duke National University Singapore, Singapore

## Abstract

**Motivation:**

Left ventricular (LV) hypertrophy is a strong predictor of cardiovascular outcomes, but its genetic regulation remains largely unexplained. Conventional phenotyping relies on manual calculation of LV mass and wall thickness, but advanced cardiac image analysis presents an opportunity for high-throughput mapping of genotype-phenotype associations in three dimensions (3D).

**Results:**

High-resolution cardiac magnetic resonance images were automatically segmented in 1124 healthy volunteers to create a 3D shape model of the heart. Mass univariate regression was used to plot a 3D effect-size map for the association between wall thickness and a set of predictors at each vertex in the mesh. The vertices where a significant effect exists were determined by applying threshold-free cluster enhancement to boost areas of signal with spatial contiguity. Experiments on simulated phenotypic signals and SNP replication show that this approach offers a substantial gain in statistical power for cardiac genotype-phenotype associations while providing good control of the false discovery rate. This framework models the effects of genetic variation throughout the heart and can be automatically applied to large population cohorts.

**Availability and implementation:**

The proposed approach has been coded in an R package freely available at https://doi.org/10.5281/zenodo.834610 together with the clinical data used in this work.

**Supplementary information:**

Supplementary data are available at *Bioinformatics* online.

## 1 Introduction

**Fig. 1.**
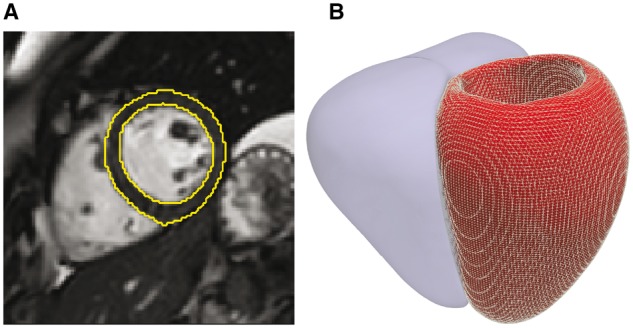
Computational image analysis. (**A**) Short axis cardiac magnetic resonance image demonstrating automated segmentation of the endocardial and epicardial boundaries of the left ventricle. (**B**) The segmentation is used to construct a three dimensional mesh of the cardiac surfaces (left ventricle shown as a mesh, right ventricle shown as a solid) that is co-registered to a standard coordinate space. Phenotypic parameters, such as wall thickness, are then derived for each vertex in the model

One of the most complex unanswered questions in cardiovascular biology is how genetic and environmental factors influence the structure and function of the heart as a three-dimensional (3D) structure (Li *et al.*, 2016). This is relevant for understanding the penetrance and expressivity of variants associated with inherited cardiac conditions as well as exploring the biology of heart development and within-population variation. Cardiac magnetic resonance (CMR) is the gold-standard for quantitative imaging (Hundley *et al.*, 2010), providing a rich source of anatomic and motion-based data, however conventional phenotyping relies on manual analysis reducing the variables of interest to global volumes and mass. Computational image analysis, by which machine learning is used to annotate and segment the images, is gaining traction as a means of representing detailed 3D phenotypic variation at thousands of vertices in a standardized coordinate space (Fig. 1) (Fonseca *et al.*, 2011; Young and Frangi, 2009).

One approach to inference is to transform the spatially correlated data into a smaller number of uncorrelated principal components (Medrano-Gracia *et al.*, 2015), however these modes would not provide an explicit model relating genotype to phenotype. A more powerful approach may be to derive a statistic expressing evidence of a given effect at each vertex of the 3D model, hence creating a so-called statistical parametric map—a concept widely used in functional neuroimaging (Friston, 2007). In this paper we extend techniques developed in the neuroscience domain to cardiovascular imaging-genetics by implementing a mass univariate framework to map associations between genetic variation and a 3D phenotype. Such an approach would provide overly conservative inferences without considering spatial dependencies in the underlying data and so we validated the translation of threshold-free cluster-enhancement (TFCE) to cardiovascular phenotypes for the sensitive detection of coherent signal (Smith and Nichols, 2009) as well as implementing robust control for multiple testing. The feasibility of the proposed methodology to derive computationally efficient inferences on imaging-genetics datasets has been tested through experiments on clinical and synthetic data using an R package developed for this purpose.

## 2 Materials and methods

### 2.1 Study population

The healthy volunteers dataset used in this study is part of the UK Digital Heart Project at Imperial College London (Bai *et al.*, 2015) (see Supplementary Data S1 for full cohort characteristic and acquisition details). To capture the whole-heart phenotype, a high-spatial resolution 3D balanced steady-state free precession cine sequence was performed on a 1.5-T Philips Achieva system (Best, the Netherlands). Images were stored on an open-source database (MRIdb, Imperial College London, UK) (Woodbridge *et al.*, 2013). Conventional volumetric analysis of the cine images was performed using CMRtools (Cardiovascular Imaging Solutions, London, UK) following a standard protocol (Schulz-Menger *et al.*, 2013).

Genotyping of common variants was carried out using an Illumina HumanOmniExpress-12v1-1 single nucleotide polymorphism (SNP) array (Sanger Institute, Cambridge). Clustering, calling and scoring of SNPs was performed using Illumina GenCall software. Samples were pre-phased with SHAPEIT (Delaneau *et al.*, 2013) and imputation was performed using IMPUTE2 (Howie *et al.*, 2009) with the UK10K dataset as a reference (www.uk10k.org). Quality of the genotypes was evaluated both on a per-individual and per-marker level using in-house Perl scripts. SNPs were removed if they had a Impute Information (INFO) score < 0.4, missing call rate in more than 1% of samples, minor allele frequency of less than 1% or deviated significantly from Hardy-Weinberg equilibrium (*P* > 0.001). Only non-related individuals with ‘CEU’ ethnicity were retained. The total genotyping rate in these individuals was 0.997 and the total number of variants available was 9.4 million.

### 2.2 Atlas-based segmentation and co-registration

All image processing was performed with Matlab (MathWorks, Natick, Mass). A validated cardiac segmentation and co-registration framework was used which has previously been described in detail (Bai *et al.*, 2015; de Marvao *et al.*, 2015). A 3D shape model (at end-diastole and end-systole) was created encoding phenotypic variation in our study population at 49 876 epicardial vertices and visualized in a standard coordinate space (Fig. 1) (Bai *et al.*, 2015). Wall thickness (WT) was measured by computing the distance between respective vertices on the endocardial and epicardial surfaces at end-diastole.

### 2.3 Overview of the approach

**Fig. 2.**
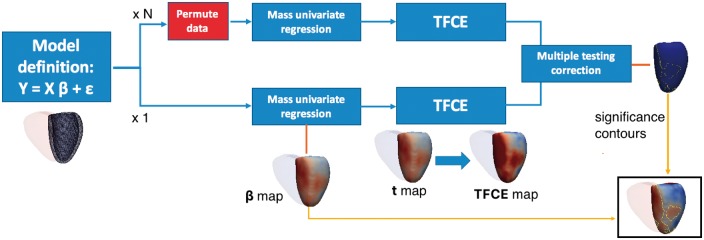
Outline of three-dimensional mass univariate framework. A statistical atlas provides point-wise measures of ventricular geometry and function which can be linked to a given predictor through a general linear model. Using mass univariate regression, three-dimensional maps of a test statistic and the degree of association (*β*) can be derived. Threshold free cluster enhancement (TFCE) coupled with permutation testing produces vertex-wise *P*-values weighted to the degree of coherent spatial support. Finally, *P*-values are corrected for multiple testing. Regression coefficients enclosed by significance contours are represented on a model of the left ventricle

In the following sections we introduce a framework for deriving associations between clinical/genetic parameters and a 3D cardiac phenotype which is outlined in Figure 2. Briefly, a general linear model is fitted at each vertex to extract the regression coefficient associated with the variable of interest (mass univariate regression). Threshold-free cluster enhancement (TFCE) is then applied to boost belief in extended areas of coherent signal in the derived vertex-wise statistical map. The points where a significant effect exists are determined by applying TFCE on the obtained t-statistic map and on *N* t-statistic maps obtained through permutation testing, derived under the null hypothesis of no effect. Then, at each vertex the frequentist probability of having obtained a higher TFCE score by chance is regarded as the *P*-value related to the regression coefficient *β*. Finally, the derived *P*-values are adjusted for multiple testing. The permutation testing procedure employed by this approach is the Freedman-Lane procedure (Freedman and Lane, 1983), whilst a false discovery rate (FDR) correction using the Benjamini-Hochberg procedure (Benjamin and Hochberg, 1995) is applied to correct for multiple testing.

### 2.4 Mass univariate analysis

The association between a ventricular phenotype mapped onto a 3D mesh and one or more clinical variables can be described using a general linear model of the form Y=βX+ϵ, where *Y* is a vector of, for example, WT values at each vertex and *X* is a design matrix that can be used to model the effect of interest and contains in each column the subject’s values of clinical co-variates as well as the intercept term. These variables can be numerical (such as age or weight), categorical or expressing interaction between them. In particular, categorical variables can be exploited to express either different categories (such as gender or ethnicity) or the presence/absence in a binary form of a clinical condition (such as the presence of genetic mutation or a specific disease). *β* is the regression coefficient vector to be estimated and ϵ represents the noise or error term, which is assumed to be a zero-mean Gaussian process and represents the variability of *Y* not explained by the model. The regression coefficient can be standardized by normalizing to mean 0 and unit-variance the columns of *X* and *Y*. As a result, *β* will represent the amount of variation of *Y* in units of its standard deviation when *X* is increased by one standard deviation, allowing comparisons between variables.

The same model can be fitted at each ventricular vertex independently (mass univariate regression) and statistics can be extracted and corrected for multiple testing in order to test one or more statistical hypotheses. In a parametric framework, the t-statistic computed as $t=\frac{\beta }{s.e.(\beta )}$ is typically used in the neuroimaging literature to contrast the null hypothesis $H_{0}:\beta =0$ (no association between the predictor and the phenotype under study), where s.e.(β) is the standard error of the estimator of *β* (Friston, 2007). The regression coefficients *β* and their related *P*-value thus obtained can be plotted to display, at high resolution on the whole 3D ventricle, the magnitude and spatial distribution of a given association. However, this approach underestimates associations where the signal is more spatially correlated than noise coherence. For this reason non-parametric statistics such as TFCE are valuable to increase the statistical power of the approach.

### 2.5 Threshold-free cluster enhancement on a cardiac atlas

The value of a statistic h obtained through mass univariate regression at a vertex p—a t-statistic in our context—is transformed by TFCE using the following integral:

(1) $$TFCE(p)=\int_{h=0}^{h_{p}}e{\left(h\right)}^{E}\;h^{H}\;\delta h≃\sum_{h=0}^{h_{p}}e{\left(h\right)}^{E}\;h^{H}\;\Delta h$$

In the equation *h_p_* is the value of the vertex statistic, *e*(*h*) is the extent of the cluster with cluster-forming threshold *h* that contains *p*, and *E* and *H* are two parameters usually set to 0.5 and 2 for empirical and analytical motivations (Smith and Nichols, 2009). In computational algorithms the integral is estimated using a discrete sum. The computational model of the heart is defined as a 3D mesh composed of non-congruent triangles where at each vertex pointwise phenotypic variables are stored for each subject. The translation of TFCE to a cardiac model is not straightforward as the model is not composed of a regular grid of voxels (as in brain imaging applications) but instead forms a mesh of vertices. We addressed this problem by associating an area to each vertex *i* equivalent to the mesh area closest to that vertex. In computing Eq. 1 at each vertex, the most time consuming part is deriving *e*(*h*)—the area of all the elements connected to p that have a statistic value greater or equal to *h*—as a different *e*(*h*) needs to be computed for each vertex of the mesh and for each term of the sum. However, the TFCE score associated to a vertex *p* of a specific *h* in the summation consists of the same score that should be associated with all the vertices which contribute to *e*(*h*). Therefore, the computational time of the TFCE method can be significantly reduced by sampling the interval between the maximum and minimum statistic *h* in the statistic map so as to use each sampled value as a threshold $\tilde{h}$ for the selection of vertices with a greater statistic value in the case of positive threshold, or less than $\tilde{h}$ in the case of a negative threshold. The edges of the graph are defined from a list containing the nearest neighbours of each vertex, and which is filtered at each iteration to contain only the vertices selected by the thresholding operation, resulting in one or more graphs of connected vertices. In this way, all the possible patterns of signal on the ventricle can be discovered without relying on assumptions about the geometry of cluster shapes. For all the obtained graphs including more than two vertices the TFCE score can be computed and associated to all the vertices that belong to them. The final TFCE score is the sum of all the TFCE scores thus obtained.

### 2.6 Permutation testing

The *P*-value associated with the regression coefficient computed at each atlas vertex can be derived via permutation testing. In particular, by permuting *N* times the input data, *N* TFCE scores maps can be obtained. It is important that the adopted permutation strategy guarantees the exchangeability assumption, *i.e.* permutations of *Y* given *X* do not alter the joint distribution of the dependent variables under the null hypothesis. In the proposed context, the *Y* values themselves are not exchangeable under the null hypothesis, as the predictors included in the model together with the variable under study are nuisance variables that could explain some portion of variability of *Y*. In order to address this problem, among a number of available techniques, the Freedman-Lane procedure (Freedman and Lane, 1983) has proved to provide the best control of statistical power and false positives (type 1 error) (Winkler *et al.*, 2014). This procedure proceeds as follows. If *Z* contains all the nuisance variables previously contained in *X*, the general linear model can be rewritten as Y=βX+γZ+ϵ. Then, instead of permuting *Y* and extracting *β*, the procedure computes the residual-forming matrix $R_{Z}=1-XX^{T}$ and performs *N* different permutations by computing the model $\hat{P_{N}}R_{Z}Y=\beta X+\gamma Z+\epsilon$ at each point, where $\hat{P_{N}}$ is the permutation operator. For a full derivation of the Freedman-Lane strategy see *Winkler et al.* (Winkler *et al.*, 2014).

### 2.7 False discovery rate correction for multiple comparisons

A multiple testing problem arises by testing tens of thousands of statistical hypotheses simultaneously. Control of the family wise error rate at 5% could be derived by extracting the maximum score from each map derived via permutation testing and by using the 95th percentile as a threshold for significance. However, in this context such a correction could be overly conservative as we are rarely interested in the exact number of vertices that reach significance. The main goal is to detect extended areas of coherent signal and therefore we can accept a maximum fixed percentage of false discoveries as provided by false discovery rate (FDR) procedures. In particular, these procedures can be applied to adjust the voxelwise *P*-values obtained at each vertex by computing the ratio between the number of times in which a TFCE score greater than the measured one has been obtained and the number of permutations *N*. We have found adaptive procedures such as the two-stage Benjamini-Hochberg (Benjamini *et al.*, 2006) not suitable for our dataset, since it led to lower *P*-values and increased areas of significance, as also reported in the neuroimaging literature (Reiss *et al.*, 2012). For this reason, the original Benjamini-Hochberg (BH) (Benjamin and Hochberg, 1995) procedure has been employed for this work. It is important to underline that both FDR correction procedures are valid when the tested hypotheses are independent or satisfy a technical definition of dependence called positive regression dependency on a subset (Benjamini and Yekutieli, 2001). This condition for Gaussian data is translated into the requirement that the correlation between null voxels or between null and signal voxels is zero or positive, and for smoothed image data as those that compose a cardiac atlas, this assumption is generally considered satisfied (Friston, 2007).

### 2.8 Software

The proposed mass univariate framework has been coded as an R package (mutools3D) which benefits from the use of vectorized operations. Matrices containing the phenotypic data and templates to visualise the 3D models are also available with the software. Linear regression assumptions must be met in order to obtain reliable inferences (for a review of them and their importance in a mass univariate setting see Supplementary Data S2). Particularly important in this context are multicollinearity and heteroscedasticity problems which should be checked and solved for each model definition. For the latter, the R package implements mass univariate functions exploiting HC4m heteroscedascity consistent estimators (Cribari-Neto and da Silva, 2011).

## 3 Results

### 3.1 GWAS replication study

As an exemplar application, six out of nine exonic SNPs which have previously shown an association with LV mass in a case-control genome wide association study (GWAS), using echocardiography for phenotyping (Arnett *et al.*, 2009), were also identified in the UK Digital Heart Project genotypes and were assessed for replication. For each SNP, WT at each vertex in the 3D model in 1124 healthy Caucasian subjects was tested for association with the posterior estimate of the allele frequency by a regression model adjusted for age, gender, body surface area (BSA) and systolic blood pressure (SBP). The tested SNPs are rs409045, rs6450415, rs1833534, rs6961069, rs10499859 and rs10483186 and cohort characteristics are reported in Supplementary Data S5. Regression diagnosis through Breush-Pagan and White’s test showed how the homoscedasticity assumption was violated at a large number of vertices, therefore mass univariate regression was corrected using HC4m heteroscedascity consistent estimators (Cribari-Neto and da Silva, 2011). Regarding the assumption of multicollinearity, the condition number of the model matrix was 2.19 while the variance inflation factor was equal to 1.06, suggesting a very low degree of multicollinearity. All the simulations were executed on a high performance computer (Intel Xeon Quad-Core Processor (30 M Cache, 2.40 GHz), 36 Gb RAM), using the analysis pipeline and R package proposed in this paper (Fig. 2). A multiple comparisons procedure correcting for the number of vertices and the number of SNPs tested was applied by simultaneously testing in a BH FDR-controlling procedure all the TFCE-derived *P*-values from all the models as suggested in (Benjamini and Yekutieli, 2005). The number of permutations was fixed to 10 000 and simulations required less than 3 h each. Finally, as a result of a preliminary study we conducted (full details in Supplementary Data S3), TFCE parameters E and H were set to 0.5 and 2, as suggested in the original TFCE paper (Smith and Nichols, 2009), since this choice provides good sensitivity and specificity on a range of synthetic signals.

**Fig. 3.**
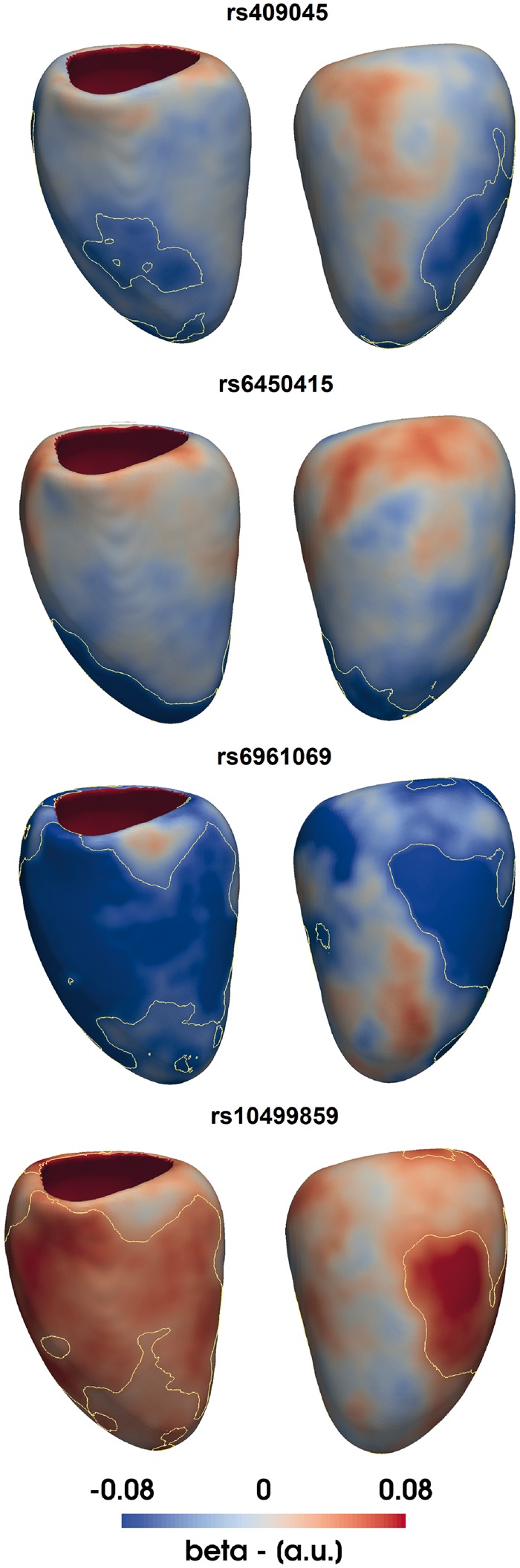
Applying three-dimensional analysis to single nucleotide polymorphism (SNP) replication. *β* coefficients are plotted on the surface of the left ventricle for the effect of 4 distinct SNPs on wall thickness (WT) adjusted for age, gender, body surface area and systolic blood pressure. Yellow contours enclose standardized regression coefficients reaching significance after multiple testing

Four SNPs showed a significant association with WT as reported in Figure 3. These are rs409045 (maximum regression coefficient $\bar{\beta }=-0.1$, percentage of the LV area significant S=13%), rs6450415 ($\bar{\beta }=-0.11,\;S=11\%$), rs6961069 ($\bar{\beta }=-0.09,\;S=44\%$) and rs10499859 ($\bar{\beta }=0.1,\;S=41\%$). Conventional linear regression analysis using LV mass and the same model for all the SNPs did not reach significance (Supplementary Data S5).

### 3.2 Assessment of sensitivity, specificity and false discovery rate on synthetic data

**Fig. 4.**
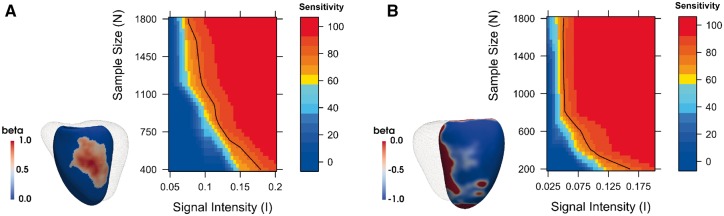
Assessment of power using synthetic data. Plots of our framework’s sensitivity at different sample sizes *N* and signal intensities *I* to detect a synthetic signal on (**A**) 10% and (**B**) 60% of the LV surface. A black line on the plots indicates a threshold of 80% sensitivity

Sensitivity, specificity and the rate of false discoveries of the proposed pipeline were estimated using synthetic data. A 3D model showing no correlation between WT and the posterior estimate of the allele frequency *X_snp_* of an non-associated SNP (rs4288653) adjusted for age, gender, BSA and SBP was used to generate background noise. A synthetic data signal was generated by summing to the WT values of each subject a term $I\;\beta \;X_{snp}$ at each vertex, where *I* is the signal intensity and *β* is a map of regression coefficients. Two contrasting *β* maps (signal A and B) obtained from real clinical data were chosen and are available in Supplementary Data S6. Signal A was characterized by non-null *β* coefficients covering the 10% of total area of the LV and scaled to the (0, 1] range, while signal B presented non-null regression coefficients scaled to the [-1, 0) range in a more extended area covering the 60% of the LV surface. By subsampling the number of subjects *N* and the signal intensity *I*, different signals to be detected by the proposed standard mass univariate pipeline were obtained. The number of permutations for each simulation was fixed to 5000 and results were linearly interpolated and plotted on the contour plots shown in Figure 4.

Sensitivity increased at larger sample sizes *N* and signal intensities *I*, reaching the greatest values with the most extended signal (signal B) as expected. Given the sample size of our GWAS replication study and the intensity of the associations found, these results would assign a sensitivity of 70% for the first two discovered SNPs and more than 90% for the other two. Moreover, the rate of false discoveries was 0 for all the results of signal A and below 5% except for few simulations involving signal B and sample sizes greater than 1600 (results reported in Supplementary Data S6). This effect is due to the large synthetic signal extension, which causes TFCE to extend its support to vertices near the true signal which show the same direction of effect. This is not considered a major limitation as TFCE will not enhance clusters that originate only from noise. Finally, sensitivity, specificity and the rate of false discoveries were also computed using our pipeline without TFCE—which showed how application of TFCE provides a relevant increase of up to 50% in sensitivity which only comes at the expense of a small decrease in specificity on large extended signals (Supplementary Data S6).

Finally, we have performed a comparative study between TFCE and a standard cluster-based thresholding method as reported in the original TFCE paper on brain imaging data (Smith and Nichols, 2009), which has been implemented in the proposed R package (Supplementary Data S4). Overall, in agreement with the neuroimaging literature, the sensitivity of the cluster-extent based thresholding method was lower than TFCE and proved to be very dependent on the cluster-forming threshold. Moreover, higher false discovery rates and lower specificities characterized cluster-extent based thresholding results in all cases when their sensitivity was comparable or greater than TFCE.

## 4 Discussion

The environmental and genetic determinants of cardiac physiology and function, especially in the earliest stages of disease, remain poorly characterized and morphological classification relies on one-dimensional metrics derived by manual image segmentation (Khouri *et al.*, 2010). In contrast, computational cardiac analysis provides precise 3D quantification of shape and motion differences between disease groups and normal subjects (Medrano-Gracia *et al.*, 2013). We have extended the application of these techniques by designing a general linear model framework that provides a powerful approach for modelling the relationship between phenotypic traits, genetic variation and environmental factors using high-fidelity 3D representations of the heart. By translating statistical parametric mapping techniques originally developed for brain mapping to the cardiovascular domain we exploit spatial dependencies in the data to identify coherent areas of biological effect in the myocardium. This framework also accounts for multiple testing correction at tens of thousands of vertices which is the main drawback of this class of techniques. In particular, the application of TFCE leads to a notable increase in power of the mass univariate approach at the expense of only a slight increase of the false discovery rate in large extended signals.

Genetic association studies using conventional 2D imaging leave much of the moderate heritability of LV mass unexplained (Fox *et al.*, 2013; Semsarian *et al.*, 2015; Vasan *et al.*, 2009). One contribution may be the lack of phenotyping power of conventional imaging metrics, which require manual analysis and are insensitive to regional patterns of hypertrophy. Our simulations on synthetic data show that our approach has the power to detect anatomical regions associated with even small genetic effect sizes. In the reported exemplar application, we replicated the effect of four SNPs discovered in a GWAS for LV mass using a 3D WT phenotype with TFCE applied, while none of the SNPs replicated with conventional LV mass analysis. The genotype-phenotype associations that we report reflect that cardiac geometry is a complex phenotype with a highly polygenic architecture dependent on anatomical patterns of gene expression and spatially varying adaptations to haemodynamic conditions (Srivastava and Olson, 2000; Wild *et al.*, 2017).

One of the main limitations of the presented framework is that high-spatial resolution CMR is not available in all cohorts, although conventional two-dimensional images may be super-resolved to provide similar shape models (Oktay *et al.*, 2017). A second limitation is that the true association may not be linear in the model parameters and nonlinear models could better fit the data. However, the advantages favouring a general linear model are its simplicity, the ability to easily design and adjust the results for multiple factors and its wide use in biomedical statistics. A third limitation of this work is with regards to the experiments using synthetic data as we only assessed noise in our single centre population and did not generalize this to other cohorts. A general limitation of these approaches is that they do not establish causal relationships, such as the interaction between genetic variants, blood pressure and LV mass, although this may be addressed in future work by Mendelian randomization.

Mass univariate approaches do not directly consider the local covariance structure of the data, however this is accounted for when Random Field Theory or permutation tests define a threshold for significant activation (Bronzino and Peterson, 2015). In the neuroimaging literature, in the context of brain-wide candidate-SNP analyses, mass univariate approaches are used more extensively than multivariate approaches as the latter are less sensitive to regional effects and they require more observations than the dimension of the response variable (i.e. number of vertices) or the use of dimensionality reduction techniques (Friston, 2007).

As the methods are computationally efficient and require no human input for phenotypic analysis, it is feasible to scale up the pipeline to larger population cohorts such as UK Biobank, which aims to investigate up to 100 000 participants using MR imaging (Petersen *et al.*, 2013). Applying these concepts to revealing the effect of rare variants on LV geometry in participants without overt cardiomyopathy (Schafer *et al.*, 2017) and to vertex-wise genome-wide analyses also represent an interesting area of future work. In this latter context, multivariate approaches may show promise for modelling high-dimensional imaging and genetic data (Liu and Calhoun, 2014; Vounou *et al.*, 2012). Finally, while we have focused on LV geometry and shape, the same approach can be applied to time-resolved vertex-wise data to create a functional phenotype for regression modelling.

## 5 Conclusion

We report a powerful and flexible framework for statistical parametric modelling of 3D cardiac atlases, encoding multiple phenotypic traits, which offers a substantial gain in power with robust inferences. We have implemented and validated the approach on both synthetic and clinical datasets, showing its suitability for detecting genotype-phenotype interactions on LV geometry. More generally, the proposed method can be applied to population-based studies to increase our understanding of the physiological, genetic and environmental effects on cardiac structure and function.

## Supplementary Material

Supplementary Figures
