# Supplementary material for: Three-dimensional cardiovascular imaging-genetics: a mass univariate framework
Source: Bioinformatics. 2017 Sep 4;34(1):97–103. doi: 10.1093/bioinformatics/btx552 (PMC5870605; doi:10.1093/bioinformatics/btx552)
Supplement: Supplementary Figures [file supplementary-data-three_btx552.pdf]

Supplementary Data for *Three-dimensional Cardiovascular Imaging-Genetics: A Mass Univariate Framework*

1 UK Digital Heart Project protocols

Participants who had known cardiovascular or metabolic disease or were taking prescription medicines were excluded but simple analgesics, antihistamines, and oral contraceptives were acceptable. Female subjects were excluded if they were pregnant or breastfeeding. Standard safety contraindications to magnetic resonance imaging were applied including a weight limit of 120 kg. All subjects provided written informed consent for participation in the study, which was approved by a research ethics committee. Anthropometric measurements were collected by trained research nurses. Subjects fasted for four hours before the visit took place. Blood pressure was acquired in accordance with the guidelines of the European Society of Hypertension (O'Brien *et al.*, 2002) using a calibrated oscillometric device (Omron M7, Omron Corporation, Kyoto, Japan). Five measures were taken, the first three were discarded and the last two were averaged. Subjects height (Ht) and weight (Wt) were assessed without shoes and body surface area (BSA) was calculated by the Mosteller formula: $BSA(m^2) = \sqrt{\frac{Ht(cm)*Wt(kg)}{3600}}$.
Cardiac MR (CMR) was performed on a 1.5-T Philips Achieva system (Best, the Netherlands). To capture the whole-heart phenotype, a high-spatial resolution 3D balanced steady-state free precession cine sequence was used that assessed the left and right ventricles in their entirety in a single breath-hold (60 sections, repetition time 3.0 ms, echo time 1.5 ms, flip angle 50°, field of view 320 × 320 × 112 mm, matrix 160 × 95, reconstructed voxel size 1.2 × 1.2 × 2 mm, 20 cardiac phases, temporal resolution 100 ms, typical breath-hold 20 s). Images were stored on an open-source database (MRIdb, Imperial College London, UK) (Woodbridge *et al.*, 2013). Conventional volumetric analysis of the cine images was performed using CMRtools (Cardiovascular Imaging Solutions, London, UK) following a standard protocol (Schulz-Menger *et al.*, 2013).

2 Gauss-Markov assumptions

In this section we review the statistical assumptions of the general linear model and their importance in a mass univariate context, so as to clarify under which conditions reliable inference can be obtained.
The regression coefficients β in the general linear model under study can be obtained via ordinary least squares (OLS): $\beta = (XX^T)^{-1}X^Ty$ and $s.e.(\beta) = \sqrt{s^2(XX^T)}$, where s^2 is the OLS estimate of the variance σ^2 of the observations. According to the Gauss-Markov theorem, the ordinary least square method will be the best linear unbiased estimator (BLUE) of the regression coefficients β if its five assumptions are satisfied. Here we report the importance of these assumptions for the approach proposed in this work.

1. *No multicollinearity in X.* Multicollinearity is present when a covariate is a linear combination (perfect) or is highly correlated (imperfect) with other covariates. The absence of perfect collinearity is necessary to guarantee that X is full rank so that XX^T can be inverted when deriving the regression coefficients, assuring their uniqueness. Imperfect multicollinearity results in a reduction of the statistical efficiency of the derived regression coefficients, causing a reduction of power and ambiguous effects (Andrade *et al.*, 1999). In order to diagnose this, low pairwise correlations between predictor variables are not sufficient to exclude multicollinearity with more than two explanatory variables, but they are a necessary condition. The variance inflation factor and the condition number of the design matrix can be instead employed to estimate the amount of variance of each regression coefficient increased because of collinearity and to detect the amount of collinearity respectively (Hair *et al.*, 2016). Taking together these three latter indices, modifications of the design matrix via omission or orthogonalization of explanatory variables can be considered to correct for multicollinearity.
2. *Random sampling of the population.* This assumption is required in order to not introduce bias in the estimates and it is guaranteed when candidates have been randomly selected to participate to the study.
3. *No endogeneity in X.* Endogeneity happens when an explanatory variable is correlated with the error term, *i.e.* $E(\epsilon_i|X) \neq 0$. In general, this problem can be due to a model misspecification problem where one or more predictors have not been included into the model, to a measurement error in X that will add bias to the estimation of the regression coefficients, or to a simultaneity error when one column of X is jointly determined with Y. As endogeneity is a conceptual problem, there are no direct statistical tests to verify this assumption, hence the results should be questioned each time. In the context under study, the last source of endogeneity can be considered negligible as imaging and clinical data comes from different sources. Bias due to measurement errors should be considered and addressed in the experimental design definition. Finally, the first assumption implies that the proposed approach can only prove association and not cause-effect relationships.

4. *Linearity and additivity.* The relation between dependent and independent variables is required to be linear in the parameters β, therefore this assumption requires the model covariates to be correctly specified. More importantly it is required that the independent variables are additive, *i.e.* the amount of change in Y associated with the increase of one predictor is independent of the values of the other covariates. This latter assumption guarantees the correct interpretation of the obtained regression coefficients, avoiding their overestimation. In our context, non-additivity can be addressed by defining interaction terms of the predictors. Moreover, when interpreting the derived regression coefficients, this assumption highlights again that this approach only shows the presence of associations and not cause-effect relationships.
5. *Homoscedasticity.* This assumption requires that the error variance σ is identical across observations. It can be tested by computing a heteroscedasticity test such as the Breusch-Pagan (for linear heteroscedasticity) or the White's test (for non-linear heteroscedasticity). Heteroscedasticity causes too wide or too narrow regression coefficient standard errors, giving too much weight to certain subsets of the data when estimating the βs. Important sources of this effect rely in physiological or artefactual effects that underlie the measurements and they can be reduced by using either using weighted least squares, log transformations of the data or heteroscedasticity-consistent robust standard errors.

If assumptions 1-4 are valid the estimation of the regression coefficients are unbiased and consistent, and if 5 is also valid it becomes efficient.

3 A study on the effect of the TFCE parameters E and H

The sensitivity of the proposed pipeline using different values of the TFCE parameters E and H were assessed using synthetic data. A 3D model showing no correlation between WT and the posterior estimate of the allele frequency X_{snp} of a SNP (rs4288653) adjusted for age, gender, BSA and SBP was used to generate background noise. A synthetic data signal was generated by summing to the WT values of each subject a term $I\ \beta\ X_{snp}$ at each vertex, where I is the signal intensity and β is a map of regression coefficients. Three distinct β maps (signal 1, 2 and 3) obtained from real clinical data and characterized by non-null β coefficients scaled to the (0,1] range were employed (Fig. **??**). These covered the 25%, 50% and 75% of total area of the LV respectively were employed together with three distinct values (0.2, 0.3, 0.4) of signal intensity I. For a given value of the signal intensity I and of the spatial extension S of the synthetic signal, five values of the parameter E and five values of the parameter H were employed by the proposed framework to detect the synthetic signal generated (a total of 25 simulations for each (I,S) couple). The number of subjects N was fixed to 80, the number of permutations for each simulation to 5,000. Sensitivity results were linearly interpolated and normalized for the maximum sensitivity obtained for each (I,S) couple and plotted on the colour plots reported in Fig. 2.

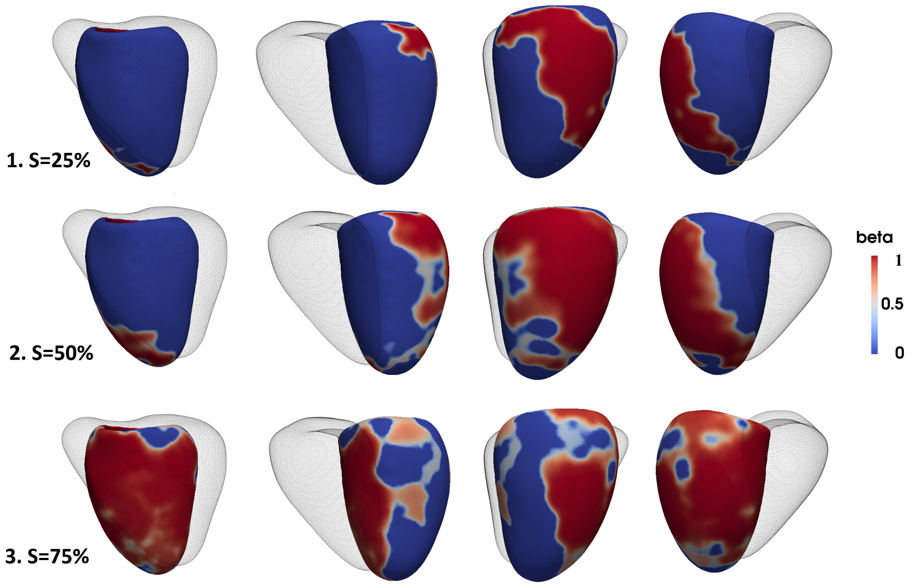

Fig. 1. The three regression coefficient β maps (signal 1, 2 and 3) used to generate the synthetic signal to be detected the method proposed in this paper with different values of the TFCE parameters E and H.

The first row of Fig. 2 shows the sensitivity results obtained at a fixed spatial extension of the generated synthetic signal ($S = 25\%$) and different signal intensities I. It can be noticed how at higher signal intensities the framework sensitivity increases and how on a small extended signal better sensitivity values are obtained when H is higher than E. The second row of Fig. 2 shows the results obtained at a same signal intensity I when increasing the spatial extension S of the generated signal. In particular, in the bottom left figure it can be noticed how the importance of the signal intensity I is still predominant, while with the increase of the signal extension S the relative importance of the parameter E increases. In all the studied cases, the false discovery rate of the framework was always below the 5% and often equal to 0%, while the sensitivity was always above 99%.
Overall, in this preliminary study the values of $E = 0.5$ and $H = 2$ suggested in the TFCE original paper (Smith *et al.*, 2009) by theoretical and empirical reasons achieved good sensitivity values. However, the performance of other combinations of E and H such as ($E = 1, H = 3$) show promise and will be investigated in future work.

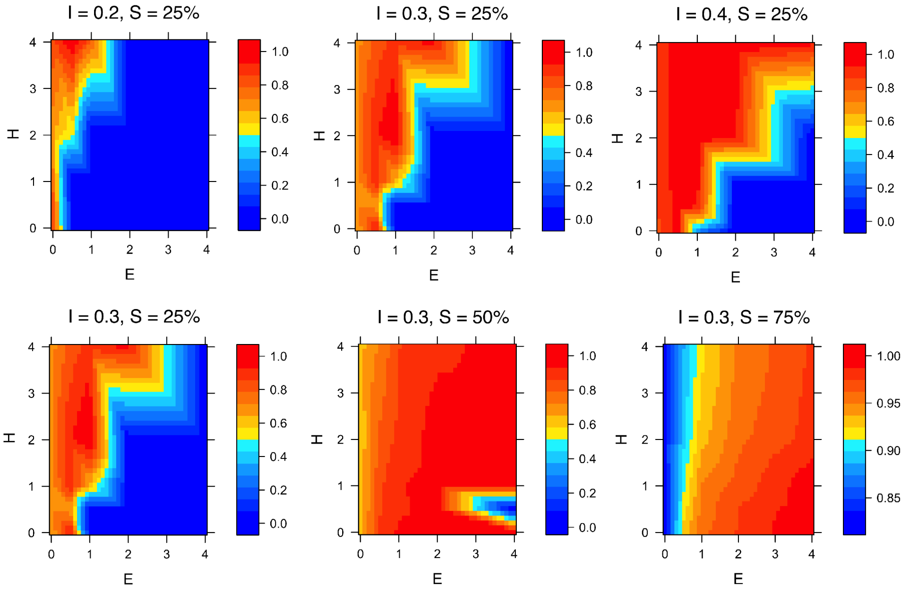

Fig. 2. Colour plots for the proposed framework sensitivity at different TFCE parameters E and H and for different signal intensities I and signal extension S. In each graph sensitivity values were normalized to the maximum sensitivity detected.

4 A comparison between standard cluster-extent based thresholding and TFCE

A comparison between the proposed framework using TFCE and the proposed framework using a standard cluster-extend based thresholding was performed on the same synthetic data used in the previous section. The latter procedure as proposed in (Friston and others., 1994) has been implemented in the R package developed for this work. This procedure consists of two steps. In the first one, a cluster in a statistical map obtained by mass univariate regression is defined as the group of connected vertices that have a t-statistic value greater than a user-defined threshold h_{thr}. Then, a second threshold h_{α} is computed via permutation testing as the 95th percentile of the distribution of largest cluster in each permuted map and used to to declare significant the clusters in the original statistical map that are more spatially extended than this threshold h_{α}. Hence this method depends on the user-defined initial cluster-forming threshold h_{thr}. For this reason, the sensitivity, specificity and FDR of the proposed approach with TFCE parameters $E = 0.5$ and $H = 2$ were compared against the results obtained by the same approach using cluster-extent based thresholding with five distinct cluster-forming thresholds h_{thr} $(0.5, 1, 1.5, 2, 2.5)$ instead of TFCE on the five distinct signals studied in the previous section (Fig. 2). The number of subjects N was again fixed to 80, the number of permutations for each simulation to 5,000 and the graphs obtained are reported in Fig. 3.
The sensitivity of the cluster-extent based thresholding method proved to be very dependent on the cluster-forming threshold h_{thr} and its choice had a large impact on the results. Moreover, higher FDR and lower specificity characterised cluster-extent based thresholding results when their sensitivity was comparable or greater than TFCE. These results therefore favour the use of TFCE over cluster-extent based thresholding as also proved in the brain image analysis literature (Smith *et al.*, 2009).

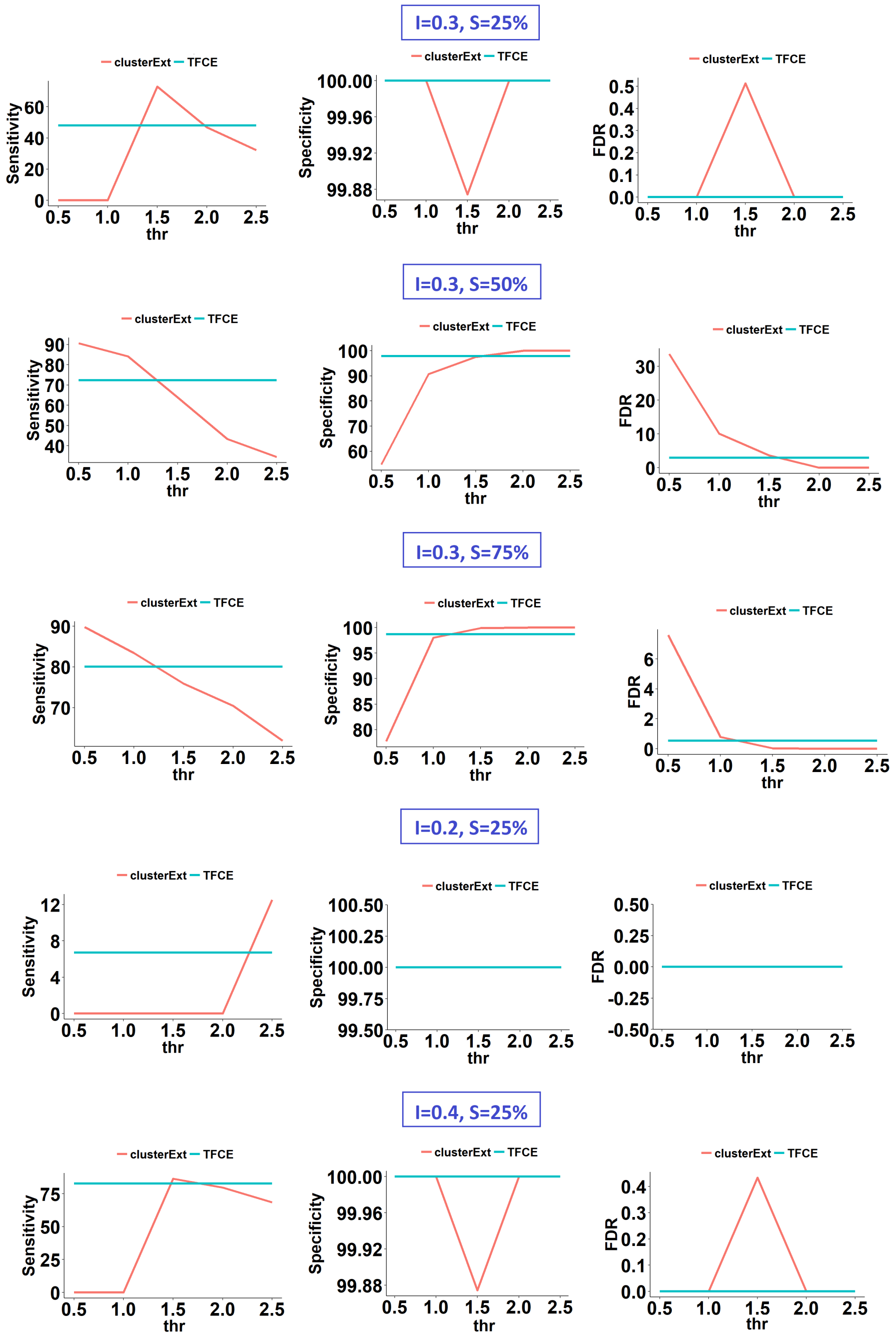

Fig. 3. Sensitivity, specificity and the false discovery rate (FDR) of the proposed pipeline using either cluster-extent based thresholding or TFCE at different cluster-forming thresholds (thr). At the top of each set of graphs, the intensity I and the spatial extension S of the generated synthetic signal is reported.

5 GWAS replication supplementary data

Table 1 contains a summary of the covariates employed in the regression model adopted fr GWAS replication. Table 2 reports the results of the linear regression models used for conventional 2D association analysis between left ventricular mass (LVM) and the posterior estimate of the allele frequency of one SNP adjusted for age, gender, body surface area (BSA) and systolic blood pressure (SBP). Even without multiple testing correction, none of the models reached significance.

	**Full Cohort** ($N = 1,124$)	**Males** ($N = 511$)	**Females**($N = 613$)
**Age** [$years$]	43.4 ± 13.3 (19-77)	43.2 ± 13.0 (19-77)	43.5 ± 13.2 (20-77)
**BSA** [m^2]	1.84 ± 0.2	1.98 ± 0.16	1.72 ± 0.14
**SBP** [$mmHg$]	119.3 ± 14	125.0 ± 12.7	114.65 ± 13.2

Table 1. A summary of the 1,124 Caucasian subjects of UK Digital Heart Project at Imperial College cohort whose MRI scans has been used in this work.

	**beta**	**p-value**
**rs409045**	0.06	0.17
**rs6450415**	0.01	0.75
**rs1833534**	-0.05	0.43
**rs6961069**	-0.01	0.96
**rs10499859**	0.01	0.84
**rs10483186**	0.01	0.74

Table 2. Regression coefficients and their related p-values of the linear association study between LVM and the posterior estimate of the allele frequency adjusted for age, gender, body surface area (BSA) and systolic blood pressure (SBP) of the presented GWAS replication study.

6 Sensitivity, Specificity and False Discovery Rate on Synthetic Data

The two regression coefficients (β) maps used to generate the synthetic data employed in this experiment are reported in Fig. 4. Signal A covers the 10% of the left ventricle and has coefficients scaled between 0 and 1, while signal B covers the 60% of the left ventricle and has coefficients scaled between -1 and 0. Fig. 5 reports the two maps obtained on signal A and B at different signal intensities (I) and sample sizes (N) for the difference between the sensitivity scored by the proposed pipeline using TFCE and the sensitivity scored by the same pipeline without TFCE. The increase of sensitivity provided by TFCE was higher for signal B due to its larger spatial extension as expected. The difference of sensitivities converged to zero at high I and N as also the sensitivity of the pipeline without TFCE reached 100% sensitivity.
Fig. 6 and Fig. 7 report the detected false discovery rate (FDR) and specificity of the proposed pipeline with or without TFCE. In Fig. 6 the FDR of the proposed pipeline was zero for all the simulated I and N, while it increased for the second signal (signal B - covering the 60% of the ventricle) and exceeded 5% only for few simulations having a sample size N greater than 1,600 (one example is reported in Fig. 7). This effect is due to the large synthetic signal extension, which causes TFCE to reward also the neighbour vertices around the true signal and it is not considered an issue since it cannot cause to declare cluster arisen from noise to be significant (as also shown in Fig. 6).
Overall, the application of TFCE provides a relevant increase in sensitivity which only comes at the expenses of a little decrease of specificity on largely extended signals.

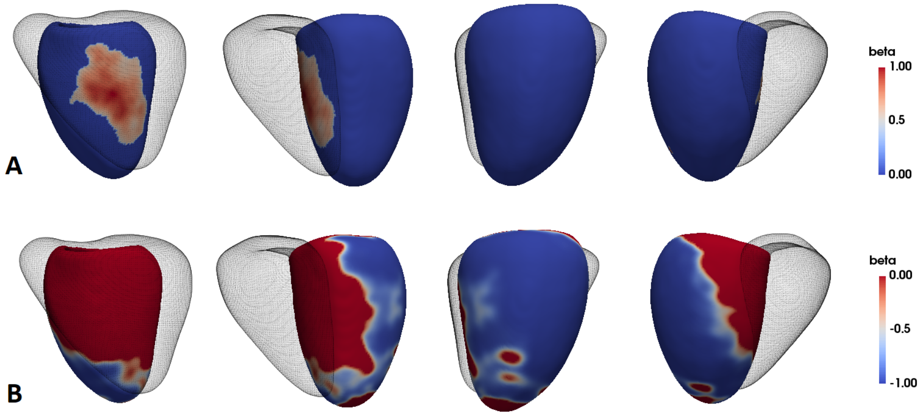

Fig. 4. The two β maps used to generate the synthetic data for this experiment - signal A (first row) and B (second row).

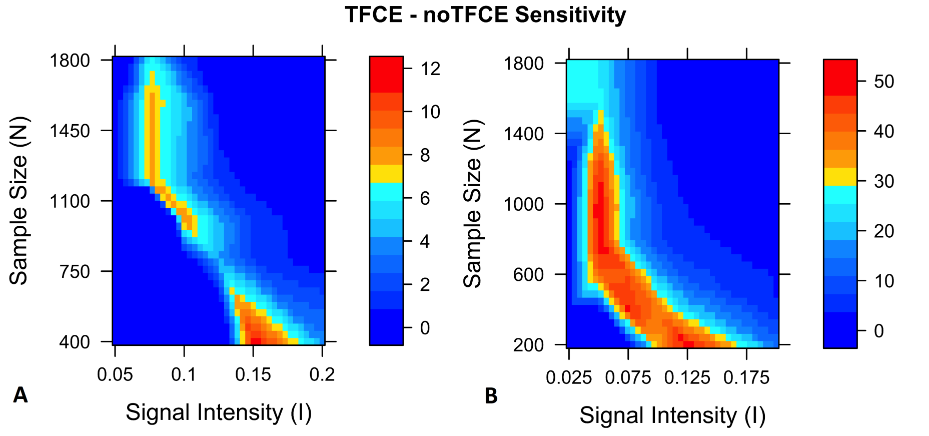

Fig. 5. 2D maps showing the increase of sensitivity of the proposed pipeline when TFCE is applied on two different synthetic signals (A and B) at different signal intensities I and cohort dimensions (N).

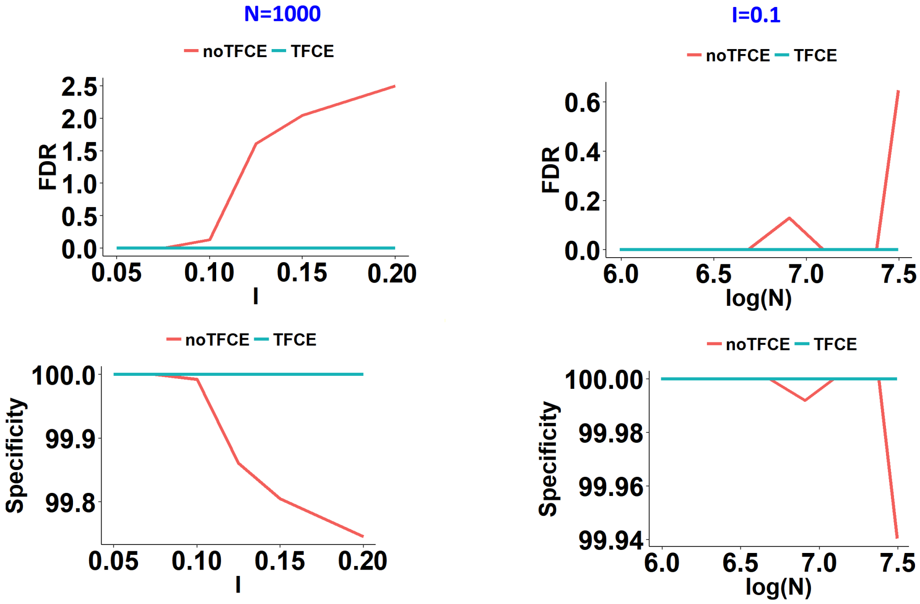

Fig. 6. Signal A. Rate of false discoveries and sensitivity of the proposed pipeline with or without TFCE.

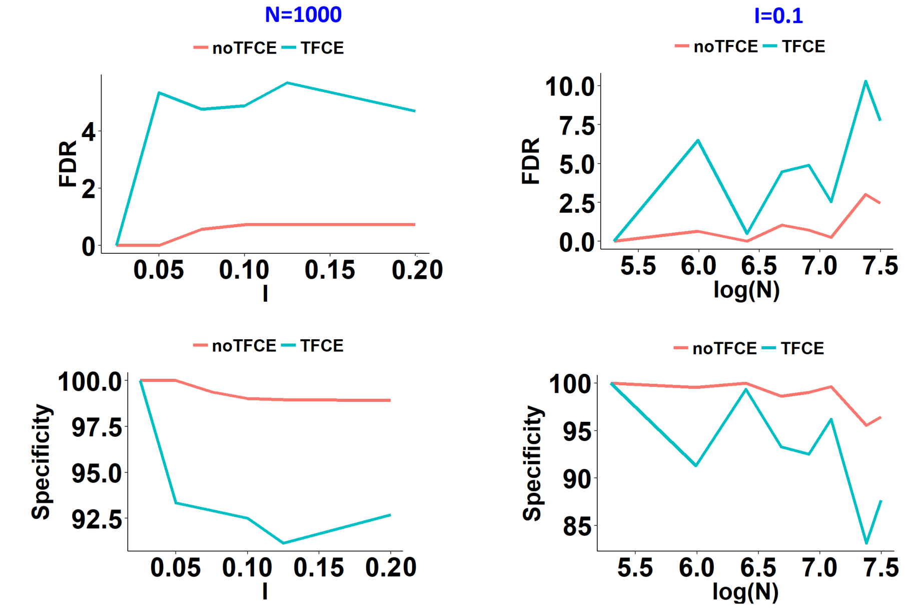

Fig. 7. Signal B. Rate of false discoveries and sensitivity of the proposed pipeline with or without TFCE.

Bibliography

Andrade, A. *et al.* (1999). Ambiguous results in functional neuroimaging data analysis due to covariate correlation. *NeuroImage*, **10**(4), 483–486.

Friston, K. and others. (1994). Assessing the significance of focal activations using their spatial extent. *Human brain mapping*, **1**(3), 210–220.

Hair *et al.* (2016). *Multivariate Data Analysis*. Pearson Education.

O'Brien *et al.* (2002). Working Group on Blood Pressure Monitoring of the European Society of Hypertension International Protocol for validation of blood pressure measuring devices in adults. *Blood Press Monit*, **7**(1), 3–17.

Schulz-Menger *et al.* (2013). Standardized image interpretation and post processing in cardiovascular magnetic resonance: Society for cardiovascular magnetic resonance (scmr) board of trustees task force on standardized post processing. *J Cardiovasc Magn Reson*, **15**(1), 35.

Smith, S. *et al.* (2009). Threshold-free cluster enhancement: addressing problems of smoothing, threshold dependence and localisation in cluster inference. *Neuroimage*, **44**(1), 83–98.

Woodbridge *et al.* (2013). MRIdb: medical image management for biobank research. *J Digital Imaging*, **26**(5), 886–890.
